# A novel expression system for production of soluble prion proteins in E. coli

**DOI:** 10.1186/1475-2859-11-6

**Published:** 2012-01-10

**Authors:** Romany NN Abskharon, Stephanie Ramboarina, Hassan El Hassan, Wael Gad, Marcin I Apostol, Gabriele Giachin, Giuseppe Legname, Jan Steyaert, Joris Messens, Sameh H Soror, Alexandre Wohlkonig

**Affiliations:** 1 VIB, Department of Structural Biology, Brussels, Belgium; 2 Structural Biology Brussels, Vrije Universiteit Brussel, Belgium; 3 Brussels Center for Redox Biology, Brussels, Belgium; 4 Department of Biochemistry and Molecular Biology, Faculty of Pharmacy Helwan University, Cairo, Egypt; 5 Laboratory of Prion Biology, Neurobiology Sector, Scuola Internazionale Superiore di Studi Avanzati (SISSA), via Bonomea 265, Trieste, Italy; 6 Italian Institute of Technology, SISSA Unit, via Bonomea 265, I-34136 Trieste, Italy; 7 ELETTRA Laboratory, Sincrotrone Trieste S.C.p.A., I-34149 Basovizza, Trieste, Italy; 8 Department of Physiology and Biophysics, Case Western Reserve University, Cleveland, Ohio 44106, USA

## Abstract

Expression of eukaryotic proteins in *Escherichia coli *is challenging, especially when they contain disulfide bonds. Since the discovery of the prion protein (PrP) and its role in transmissible spongiform encephalopathies, the need to obtain large quantities of the recombinant protein for research purposes has been essential. Currently, production of recombinant PrP is achieved by refolding protocols. Here, we show that the co-expression of two different PrP with the human Quiescin Sulfhydryl OXidase (QSOX), a human chaperone with thiol/disulfide oxidase activity, in the cytoplasm of *E. coli *produces soluble recombinant PrP. The structural integrity of the soluble PrP has been confirmed by nuclear magnetic resonance spectroscopy, demonstrating that properly folded PrP can be easily expressed in bacteria. Furthermore, the soluble recombinant PrP produced with this method can be used for functional and structural studies.

## Introduction

Prion diseases, also referred as transmissible spongiform encephalopathies (TSEs), are a family of rare progressive neurodegenerative disorders that affect both humans and animals [1]. TSEs include, for instance, bovine spongiform encephalopathy (BSE) in cattle, and Creutzfeldt-Jakob disease (CJD) in humans. These disorders are characterized by long incubation periods and characteristic spongiform changes in the brain associated with neuronal loss. The causative agent of TSEs is an infectious protein known as prion (also denoted as PrP^Sc^) [2]. This pathogenic beta-sheet-rich conformer derives from the normal, mostly alpha-helical isoform, cellular prion protein (PrP or PrP^C^), through a conformational conversion event which leads to aggregates in the brains of affected individuals leading to neurodegeneration [3].

Since the identification of prions as the causing agent of TSEs, recombinant PrP has been instrumental to study the structural and biophysical aspects of prion amyloidosis [4].

Due to its easy handling, inexpensive medium and large-scale production, the enteric bacterium *Escherichia coli *(*E. coli*) is the organism of choice for the production of numerous recombinant proteins [5]. However, expression of mammalian proteins in *E. coli *remains difficult and often results in inactive aggregates because the recombinant proteins do not fold properly in this host [6]. For example, recombinant PrP is largely expressed as inclusion bodies [7]. Most refolding protocols require a large amount of reagents and are time consuming. Success is highly dependent on the experimenter's *savoir-faire*. Attempts to assist proper folding of the PrP in the cytoplasm of *E. coli *by co-expression with bacterial chaperones failed [8]. As the formation of a disulfide bond is essential for PrP proper folding, expression of full-length PrP in the periplasm of *E. coli *was also investigated, resulting in soluble PrP which is partially degraded at the unstructured N-terminal end [9]. It has been observed that PrP can interact with several chaperones from the endoplasmic reticulum (ER) [10], including Pdia3 (also known as ERp57) and Grp58 (ERp60) [11], suggesting that in physiological condition, PrP requires assistance to fold into the correct conformation. In addition, PrP contains a disulfide bond which is crucial for the proper α-helical fold [12]. Based on these observations, we investigated the use of QSOX as a folding catalyst for PrP in the cytoplasm of *E. coli*. QSOX is a human chaperone that introduces disulfide bonds in secreted proteins downstream of the ER [13], and has been shown to be enzymatically active in the bacterial cytoplasm [14].

In the present study, we describe for the first time the production of soluble PrP of both mouse (MoPrP) and human (HuPrP) using co-expression with QSOX in the *E. coli *cytoplasm.

## Materials and methods

### Plasmids Construction

#### Human PrP (HuPrP): full length (23-231) and truncated (90-231)

Cloning of HuPrP(90-231) into pET-28a (Novagen) was performed as described previously [15]. HuPrP(23-231) was subcloned from the plasmid HuPrP(23-231)/pET-11a [16] into pET-28a as *BamHI*-*NdeI *fragment using the standard molecular biology techniques.

#### Mouse PrP(MoPrP): full length (23-230) and truncated (89-230)

Cloning of MoPrP(23-230) and MoPrP(89-230) as described previously [15].

### QSOX

The human *QSOX *plasmid was a generous gift from Prof. Colin Thorpe [13].

### Protein expression

#### Small-scale expression trials

Each PrP construct and the QSOX plasmid were co-transformed into *E. coli *Rossetta (DE3) pLysS and plated on LB-agar supplemented with 100 μg/mL ampicillin and 25 μg/mL kanamycin. A single colony was used to inoculate a 25 mL pre-culture (LB medium supplemented with 100 μg/mL ampicillin and 25 μg/mL kanamycin). The following day, a 25 mL culture was inoculated with 1 mL of the pre-culture. Cells were induced at A_600 _= 0.7 by adding 1 mM isopropyl-b-D-thiogalactopyranoside (IPTG). After induction culture was incubated overnight (16 h) at 15°C. Harvested cells were resuspended in lysis buffer: 0.1 g of cell paste/mL of 50 mM potassium phosphate, pH 7.5, 300 mM NaCl supplemented with 0.1 mg/mL lysozyme, 0.1 mg/mL ABESF and 1 μg/mL leupeptin. The lysate was sonicated 4 times, each 30 s at 4°C and was subsequently centrifuged 20 min at 18,000 g. Supernatant was collected and pellet was resuspended in initial volume using lysis buffer. Total fraction, supernatant and pellet were analyzed for the presence of soluble PrP by SDS/PAGE and immunoblotting.

### Growth curve and quantification of PrP production

For plotting the growth curve of MoPrP(89-230) small-scale cultures (MoPrP/QSOX and MoPrP/no QSOX) were produced as described previously. Samples were collected at 30 minutes time intervals and *A*_600 _was measured. Optical density was plotted versus time.

To quantify the amount of PrP produced at the different times during the growth, 1 L culture was induced as described earlier and 40 mL samples were collected at 0, 1, 2, 4, 8 and 16 h after induction. Cells were collected by centrifugation at 15,000 g for 10 min, weighted and resuspended in (0.1 g of cell paste/mL) volume of lysis buffer to normalize the cell content for each time point. For estimating the total prion production 4 μl were mixed with 1 μl SDS loading buffer (5X) and boiled for 5 min. To determine the quantity of soluble expressed PrP, resuspended cells were lysed by sonication and centrifuged at 18,000 g for 20 min. 4 μl of supernatant was mixed with 1 μl SDS loading buffer (5X) and boiled for 5 min. Collected samples were analyzed on SDS-PAGE and by immunoblotting. Intensity of the blot signals was quantified using the software LabImage 1D Gel Analysis (Kapelan GmbH, Germany).

### Immunoblotting

Mouse monoclonal anti-His antibody was purchased from Sigma Aldrich, Belgium. Proteins analyzed on SDS-PAGE were transferred to nitrocellulose membranes (MACHEREY-NAGEL) and bands were visualized by goat anti-mouse IgG, alkaline phosphatase conjugate (Sigma) using NBT/BCIP as substrate (Roche Diagnostics, GmbH, Germany).

### Large-scale protein expression and purification

MoPrP(89-230), HuPrP(90-231), MoPrP(23-230) and HuPrP(23-231) were co-expressed with QSOX in *E. coli *Rossetta (DE3) pLysS. Expression up-scaling was carried out as follows. Pre-cultures (25 mL) were grown overnight at 37°C in LB medium supplemented with 100 μg/mL ampicillin and 25 μg/mL kanamycin. 10 mL of pre-culture were used to inoculate 1 L of LB medium supplemented with ampicillin and kanamycin. Cells were induced at A_600 _= 0.7 by adding 1 mM isopropyl-b-D-thiogalactopyranoside (IPTG) and temperature was shifted to 15°C for 16 h.

Cells were harvested by centrifugation (15 min at 15,000 g). Cells pellets were re-suspended as 0.1 g of cell paste/mL in 50 mM potassium phosphate, pH 7.5, 300 mM NaCl supplemented with 0.1 mg/mL lysozyme, 0.1 mg/mL AEBSF and 1 μg/mL leupeptin. Cells were disrupted two times with a French press (10,000 psi) and followed by centrifugation at 4°C for 60 min at 40,000 g. The collected supernatant was loaded on a 5 mL Histrap Ni-NTA column (GE-healthcare) previously equilibrated with 50 mM potassium phosphate pH 7.5, 300 mM NaCl, 10 mM imidazole. The column was washed with five column volumes (CV) of washing buffer: 50 mM potassium phosphate pH 7.5, 1 M NaCl, 50 mM imidazole, followed by ten CV of 50 mM potassium phosphate pH 6.0, 1 M NaCl, 50 mM imidazole. The protein was eluted with a gradient of imidazole from 50 mM to 1 M in 50 mM potassium phosphate pH 7.5. The elution peak fractions were loaded on a SDS/PAGE to evaluate purity, then pooled, and concentrated for a second purification step. Size exclusion chromatography was performed in 20 mM Tris-HCl pH 7.5 with 150 mM NaCl on a Superdex75 HR 10/30 (GE Healthcare). The elution peak was again loaded on SDS/PAGE and collected for a dialysis against 10 mM sodium acetate pH 4.6, 1 mM EDTA followed by the final dialysis buffer of 10 mM sodium acetate pH 4.6. Protein aliquots were stored at -80°C until further usage.

### Circular dichroism (CD) experiments

CD spectra were recorded at 25°C using a spectropolarimeter (Jasco, model 715, Tokyo, Japan). The purified protein was diluted in water to the final concentration of 0.2 mg/mL. CD spectra were acquired at a scan speed of 50 nm/min, 1 nm bandwidth and a response time of 1 s. A 0.01 cm path length quartz cells was used to record spectra of the different proteins in the far ultraviolet region (190-260 nm), each spectrum was recorded 4 times. The sample and the buffer solutions were purged with dry nitrogen.

### Nuclear magnetic resonance (NMR) measurements

Uniformly ^15^N and both ^15^N and ^13^C-labeled MoPrP(89-230) were produced in M9 minimal medium supplemented with ^15^NH_4_Cl and [^13^C] glucose as described by Marley et al. [17]. The labelled proteins were purified as described above for unlabelled proteins.

The NMR experiments were recorded on ^15^N, ^13^C-labeled MoPrP(89-230) concentrated to approximately 0.5 mM into 10 mM sodium acetate buffer pH 4.6. Two dimensional NMR ^15^N and ^13^C heteronuclear single quantum correlation (HSQC) spectra and three dimensional NMR experiments CBCA(CO)NH, HNCACB, HNCO, HBHA(CO)NH were performed at 293 K on MoPrP(89-230) on a Varian NMR Direct-Drive Systems 800 MHz spectrometer equipped with a salt tolerance triple-resonance PFG-Z cold probe. Sequence specific backbone ^1^HN, ^15^N, ^13^C', ^13^C^a^, ^13^C^b^, H^a ^and H^b ^chemical shifts were determined using standard triple-resonance assignment methodology [18]. Identical experiments were performed for MoPrP(89-230) at pH 7.0. All the NMR data were processed with NMRPipe 2.1 software [19] and analysed using CCPNmr Analysis 2.0 [20]. CS23D2.0 [21] was used to generate a 3D model of MoPrP(89-230) at pH 4.6 using only the backbone chemical shifts.

### Thiostar assay

The thiostar assay determines accurately the amount of free thiol content in samples [22]. To perform this assay, we used the kit Detect X™, Luminos (Arbor assays, USA). A standard curve was plotted using reduced L-glutathion (Sigma, Belgium) at different concentrations (0 μM, 10 μM, 20 μM, 30 μM, 40 μM, 50 μM and 60 μM). Two protein samples were tested: soluble PrP and refolded, both samples were tested at a fixed concentration of 5 μM. All standards and samples were diluted 10 times in a thiol-free buffer to a final volume of 100 μL in a 96-well plate. Then we added 15 μL of Thiostar reagent to each well. After mixing, we incubated the plate at room temperature for 30 min in the dark. The fluorescent product was measured at 510 nm in a fluorescent plate-reader (Infinite M200, TECAN) with excitation at 390 nm.

### Amyloid seeding assay (ASA) protocol

MoPrP(89-230) has been diluted to 0.1 mg/mL in phosphate-buffered saline (PBS) solution containing 0.4 M Guanidine hydrochloride (GuHCl), 10 mM Thioflavin T (ThT). The fibrilization reaction was performed in a final volume of 200 μL in 96-well plate (BD Falcon, BD Bioscience). For seeding experiment we performed the amyloid seeding assay (ASA) according to Colby et al., 2007 [23] with some minor modifications. Briefly, 1 mg of ScGT1 cell lysate were used for PTA precipitation by adding 500 μL of PBS containing 4% sarkosyl, protease inhibitor (Complete, Roche) and 0.5% PTA, with continuous shaking at 37°C, 350 rpm for 1 h and centrifuged 14,000 g for 30 min. The pellet was washed and resuspended in the previous buffer, then centrifuged again and re-suspended in 150 μL of sterile double distilled H_2_O. In ASA, 4 μL of re-suspended PTA pellet were diluted in 400 μL of water and 20 μL of diluted sample were added to each well. The plate was incubated at 37°C with continuous shaking on a plate reader (Spectramax M5, Molecular Device). The kinetics of fibril formation was monitored by top reading of fluorescence intensity every 5 min at 444 nm excitation and 485 nm emission.

### Conversion of the monomeric MoPrP into amyloid fibrils

MoPrP(89-230) was diluted into 50 mM phosphate buffer, 2 M GuHCl, pH 6.5 to a final concentration of 0.4 mg/ml and incubated at 37°C with constant rotation (8 rpm).

### Atomic Force Microscopy (AFM)

The converted PrP samples were incubated on a freshly cleaved mica surface for approximately 30 seconds and subsequently rinsed with water to remove salts and unbound protein. After drying the samples were imaged using tapping mode on a Digital Instruments Multimode atomic force microscope equipped with Nanoscope IV controller and a type E scanner. All images were acquired using single-beam silicon probes with a nominal spring constant of 40 N/m and nominal tip radius of 10 nm.

### X-ray fiber diffraction

Converted PrP was pelleted by centrifugation at 16,000 g, and pellet was subsequently washed twice with water to remove salts. The wet pelleted fibrils were pipetted in a 2 mm space between two fire-polished glass rods and allowed to dry. Diffraction patterns were collected using a Rigaku MicroMax-007HF copper anode X-ray source and a Rigaku Saturn 944+ CCD detector.

## Results and discussion

**Figure 1:**
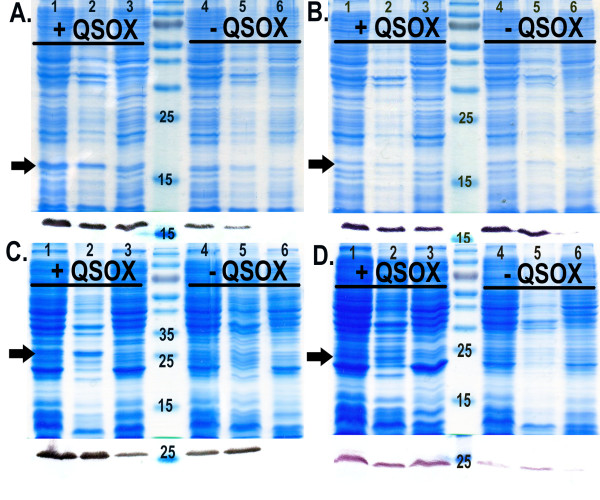
**Effect of the co-expression of QSOX on the expression of soluble PrP in the cytoplasm of *E. coli***. A) SDS-PAGE analysis of MoPrP(89-230). Expression trials in presence of QSOX: Lane 1 = total *E. coli *lysate, Lane 2 = insoluble fraction, Lane 3 = soluble fraction. Expression trials in absence of QSOX: Lane 4 = total *E. coli *lysate, Lane 5 = insoluble fraction, Lane 6 = soluble fraction. The black arrow indicates the MoPrP(89-230). The corresponding immunoblot using anti-His-tag antibody is shown underneath the SDS-PAGE gel. B) SDS-PAGE and immunoblot analysis of HuPrP(90-231). C) SDS-PAGE and immunoblot analysis of MoPrP(23-230). D) SDS-PAGE and immunoblot analysis of HuPrP(23-231).

**Figure 2:**
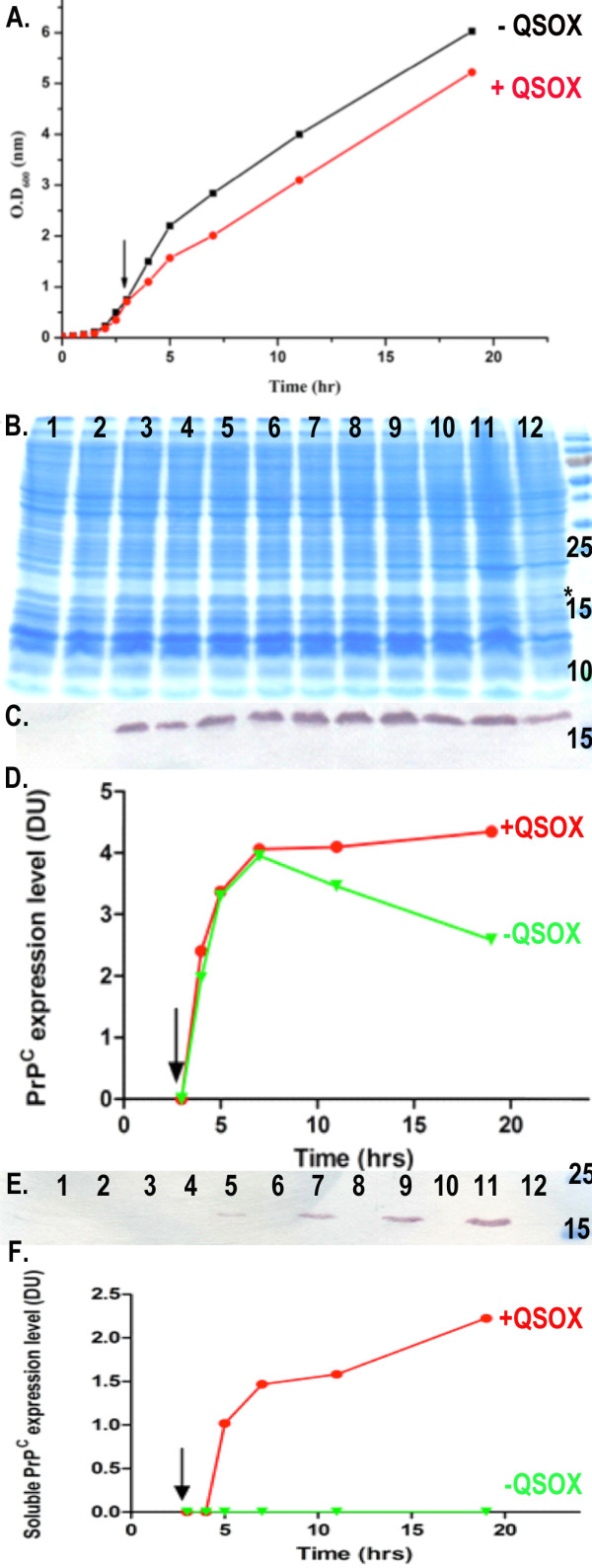
**Production of MoPrP(89-230) in *E. coli***. A) Growth curve of *E. coli *cells, showing the expression of MoPrP(89-230): absence of QSOX and co-expressed with QSOX in black and in red respectively. B) SDS-PAGE of total cell extract. Lane 1: (+) QSOX before induction, lane 2: (-) QSOX before induction, 3: (+) QSOX 1 h after induction, lane 4: (-) QSOX 1 h after induction, lane 5: (+) QSOX 2 h after induction, lane 6: (-) QSOX 2 h after induction, lane 7: (+) QSOX 4 h after induction, lane 8: (-) QSOX 4 h after induction, lane 9: (+) QSOX 8 h after induction, lane 10: (-) QSOX 8 h after induction, lane 11: (+) QSOX 16 h after induction, lane 12: (-) QSOX 16 h after induction and lane 13: MW. C) Immunoblot showing the expression level of total MoPrP(89-230) over time. D) Quantification of the total production of MoPrP(89-230) expressed over 16 h. Intensities from the western blot signal were quantified using the LabImage software, in presence of QSOX and in absence of QSOX in red and green respectively. E) Immunoblot showing the expression level of soluble MoPrP(89-230) over time (same time points as in B). F) Quantification of the production of soluble MoPrP(89-230) expressed over 16 h, in presence of QSOX and in absence of QSOX in red and green respectively.

**Figure 3:**
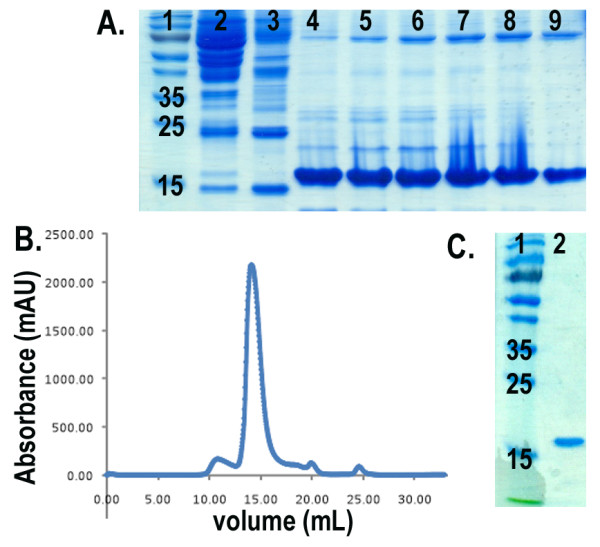
**Purification of soluble MoPrP(89-230)**. A) Purification of MoPrP(89-230) after Ni-NTA. Lane 1: Molecular weight. Lane 2: Lysate (soluble fraction). Lane 3: Flow through. Lane 4 to 9: Elution peak. B) Size exclusion chromatogram (Superdex75 HR10/30). C) SDS/PAGE of pooled fractions from the highest elution peak (from 13 to 16 mL elution volume). Lane 1: Molecular weight. Lane 2: pooled fractions contain purified PrP.

Two set of constructs for each species were used to evaluate the effect of QSOX co-expression in increasing the solubility of PrP: full-length MoPrP(23-230), HuPrP(23-231), truncated MoPrP(89-230) and HuPrP(90-231). The constructs were cloned into pET28a plasmid, while the QSOX gene was expressed in a pTRC-HisA vector. Both plasmids are IPTG inducible for simultaneous expression of both proteins. We evaluated the expression level and solubility of each construct on SDS-PAGE and immunoblot with anti-His-tag antibody (Figure 1). In addition, we examined protein expression and solubility of MoPrP(89-230) in the presence and absence of QSOX at various time points after induction in standard LB broth. After induction, cells expressing both proteins showed a reduced growth rate compared to the one expressing only MoPrP(89-230) (Figure 2A), which is probably an effect of the metabolic burden imposed by the double recombinant proteins over-expression [24]. Immunoblot analysis using anti-His-tag specific antibodies on total cell extracts showed that the total production of MoPrP(89-230) during the first three hours of the induction is equivalent between cells with or without QSOX. However, after three hours of induction a difference in total PrP production between both cells is observed. In cells without QSOX, the total concentration of PrP decreased compared to cells with QSOX, where the PrP concentration remained constant over time (Figure 2B, C, D). Expression of PrP without QSOX is known to produce insoluble recombinant protein [8], hence the decrease in PrP concentration could be attributed to the degradation of the insoluble fraction by cytoplasmic proteases [25]. We further estimated the quantity of soluble MoPrP(89-230) during the course of the induction (Figure 2E, F). In absence of QSOX, no soluble PrP is present in the cell extracts throughout the 16 hours of induction, while in presence of QSOX, soluble PrP can be detected after two hours induction. The quantity of PrP in the soluble fraction increased until it reached a plateau after 16 hours of induction. The soluble recombinant PrP has been purified by Immobilized Metal Affinity Chromatography (IMAC), followed by a size exclusion chromatography to high purity (Figure 3). The yields per litre of culture after purification were 1 mg/L for MoPrP(23-230) and 9 mg/L for MoPrP(89-230). For the human PrP, the yield was 1.5 mg/L for HuPrP(23-231) and 3 mg/L for HuPrP(90-231). Attempts to produce soluble PrP with commercially available chaperones like DnaK, DnaJ, GrpE, GroES, GroEL failed (data not shown).

**Figure 4:**
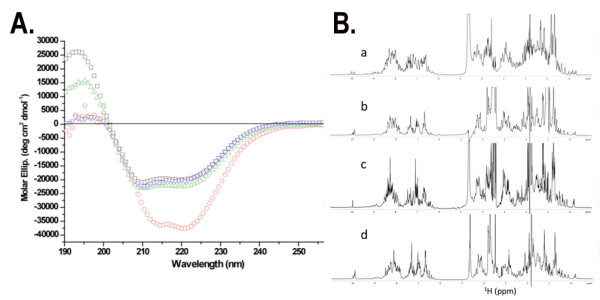
**Biophysical characterisation of the four soluble PrP**. A) Far-UV CD spectra of the four soluble PrP collected at 25°C in sodium acetate buffer pH 4.6. MoPrP(23-230) in red, HuPrP(23-231) in blue, MoPrP(89-230) in black and HuPrP(90-231) in green. B) 1D 800 MHz ^1^H-NMR spectra of the soluble PrP: (a) MoPrP(89-230), (b) MoPrP(23-230), (c) HuPrP(90-231) and (d) HuPrP(23-231). All spectra were recorded at 25°C, pH 4.6.

**Figure 5:**
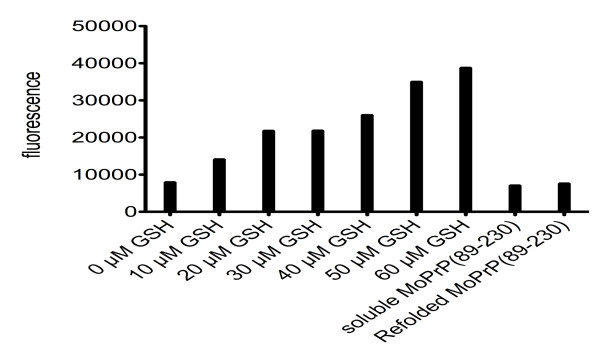
**Thiostar assay for soluble and refolded MoPrP(89-230)**. Fluorescence of free thiols in soluble and refolded PrP (5 μM) compared to reduced L-glutathione (0 μM, 10 μM, 20 μM, 30 μM, 40 μM, 50 μM and 60 μM).

In the next step, we evaluated the structure of the different PrP construct co-expressed with QSOX with far-UV circular dichroism (CD) and nuclear magnetic resonance (NMR) spectroscopy. All CD spectra show a double minimum at 208 and 222 nm, characteristic for α-helical proteins, indicating that all four PrP contain a significant amount of secondary structure (Figure 4A). Moreover, the 1D ^1^H NMR spectra showed that all soluble expressed PrP are well folded (Figure 4B). Previous studies on PrP showed that the presence of a disulfide bond is essential to maintain the α-helical fold of the C-terminal end [12]. Therefore, we verified whether the disulfide is formed in soluble expressed PrP using a Thiostar assay [22] (Figure 5). No free thiols were found in soluble expressed PrP, confirming that a disulfide bond is indeed formed upon expression in the cytoplasm.

**Figure 6:**
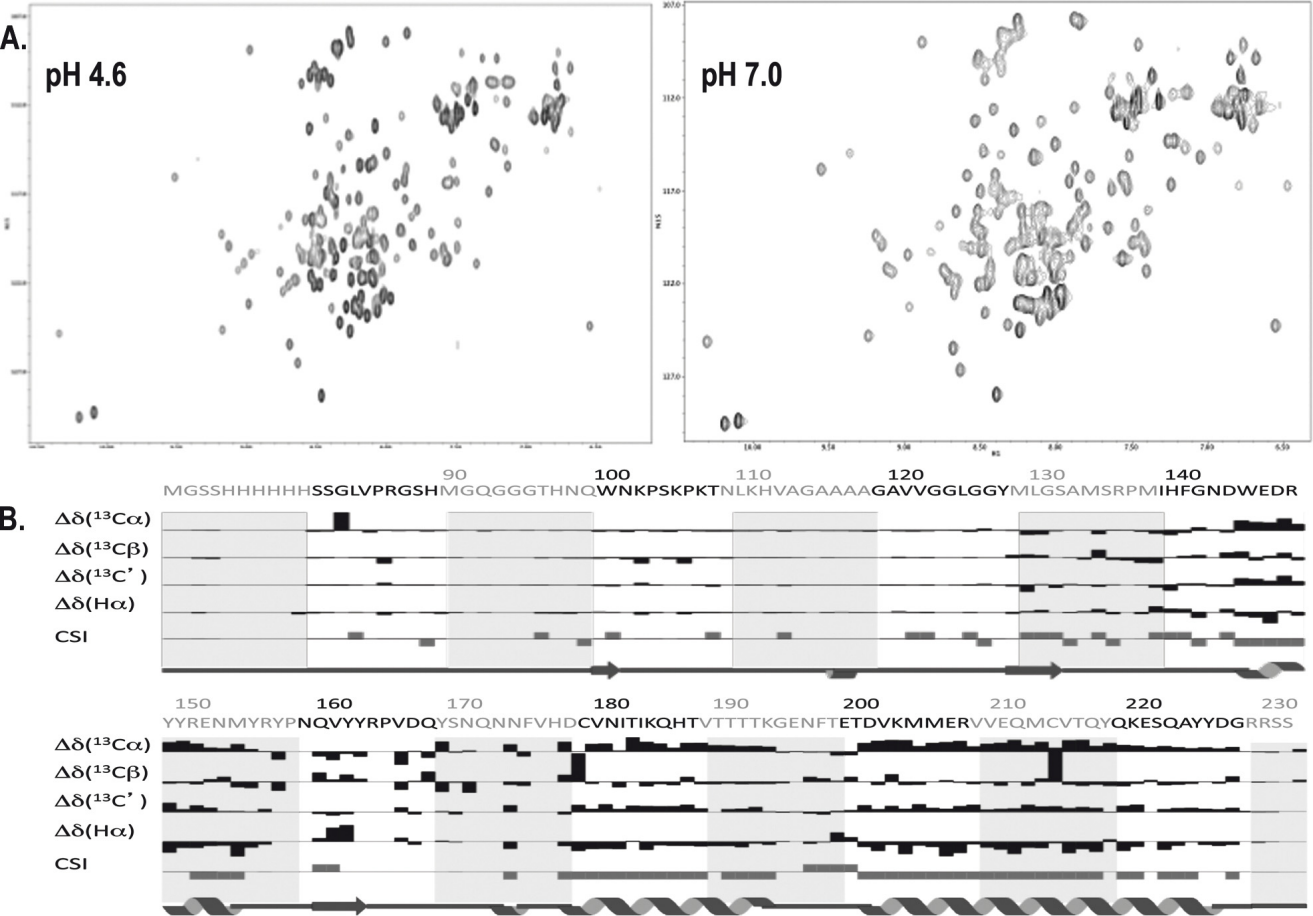
**NMR spectra and Chemical Shift Index for MoPrP(89-230)**. A) 800 MHz ^1^H-^15^N HSQC at pH 4.6 and pH 7.0 recorded at 25°C. B) Chemical Shift Index (CSI) at pH 4.6 with the secondary structure prediction based on the backbone chemical shifts. The primary sequence of MoPrP(89-230) is shown on the top.

**Figure 7:**
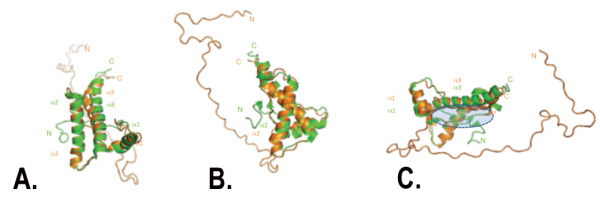
**Structural superposition of the refolded and soluble MoPrP(89-230) 3D structures**. Superimposition was generated with CS23D2.0 based on the backbone chemical shifts in green and orange respectively shown in two different orientations (A and B). The three helices α1, α2 and α3 are annotated for each structure. C) The structure of the refolded MoPrP(121-230) coloured in green exhibits a β-sheet between 128-130 and 161-163. Although this β-sheet is not present in the model of MoPrP(89-230) generated with CS23D2.0, the region Y156-Q167 that includes the second β-strand 161-163 in the refolded MoPrP(121-230) is well-superimposed with that in MoPrP(89-230) as highlighted by the blue circle.

**Figure 8:**
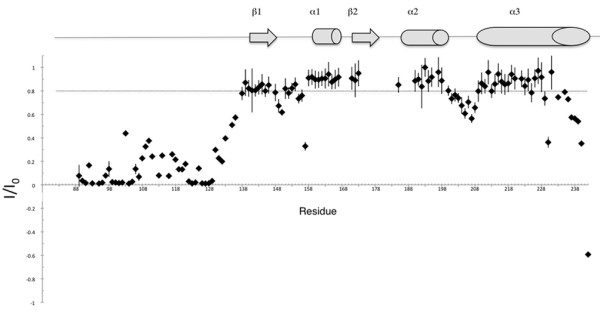
**Plots of the ^1^H-^15^N NOEs versus the residue numbers of MoPrP(89-230)**. A schematic of the secondary structure of MoPrP(89-230) is shown above the graph.

In order to explore in detail the structure of the soluble mouse PrP, uniformly ^15^N-^13^C-labelled MoPrP(89-230) was produced in co-expression with QSOX. ^1^H-^15^N HSQC spectra are characteristic for a well-folded protein with no tendency for aggregation at both pH 4.6 and pH 7.0 (Figure 6A). Backbone assignments at pH 4.6 are essentially complete (≈ 96%), except for the ten N-terminal residues that include the hexahistidine tag. Most importantly, the Cβ chemical shifts of Cys179 and Cys214 are indicative for a disulfide bond between the two cysteines, concurring with the Thiostar assay. The consensus chemical shift index (CSI) [26] indicates the presence of three α-helices (144-154), (177-193), and (200-227). The CSI also suggests that regions (128-131) and (160-164) show some propensity for β-strand conformation (Figure 6B). A preliminary three-dimensional model of MoPrP(89-230) was generated from the ^1^H, ^13^C, and ^15^N backbone chemical shifts using CS23D2.0 [21]. The resulting three-dimensional model exhibits mainly an α-helical structure with three α-helices displaying an orientation similar to the previous structure of MoPrP(121-231) [27]. Alignment of the two structures suggests that α2 and α3 helices in our model are slightly more elongated (Figure 7). Interestingly, α3 in our model forms a regular α-helix from E200 to G227, with no break between Q219 and S222 as observed in the NMR structure of the refolded MoPrP(121-231) [27]. ^1^H-^15^N heteronuclear NOEs measured in MoPrP(89-230) show no significant variations within the (200-227) region with an average value of 0.8 indicative of restricted motions along the α3 helix (Figure 8). The structure of the refolded MoPrP(121-231) exhibits a β-sheet between 128 to 130 and from 161 to 163 [27]. Although this β-sheet is not proposed in the model of MoPrP(89-230) generated with CS23D2.0, manual analysis of ^15^N-NOESY-HSQC and ^13^C-NOESY-HSQC spectra allowed the identification of specific long range NH-NH and CH^α^-CH^α ^NOEs between the residues 127 to 130 and 161 to 164 (data not shown) supporting the formation of the antiparallel β-sheet present in the soluble MoPrP(89-230) protein.

**Figure 9:**
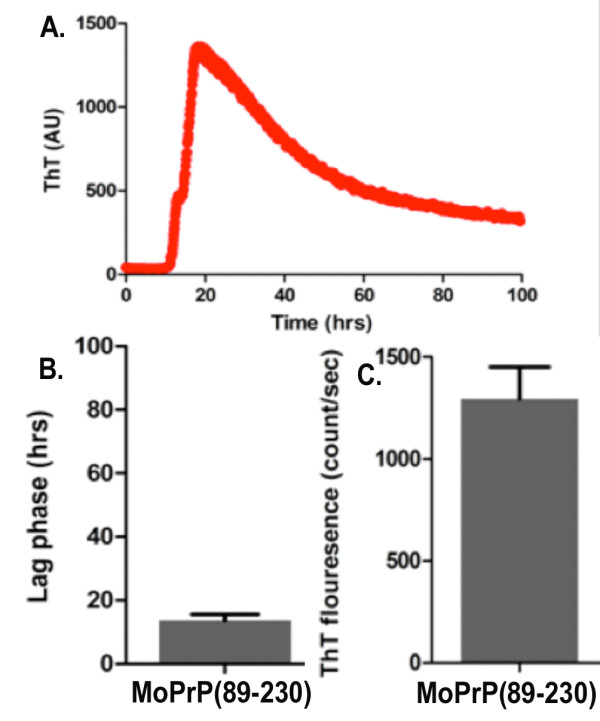
**Kinetics of MoPrP(89-230) produced in the ASA**. A) Kinetic of ThT positive MoPrP(89-230) aggregate. This sigmoidal curve is typical of an increased content of β-sheet structures together with enhanced aggregation of MoPrP(89-230). B) Mean values of the lag phase. C) Mean value of the maximum ThT intensity under native condition (n = 4).

**Figure 10:**
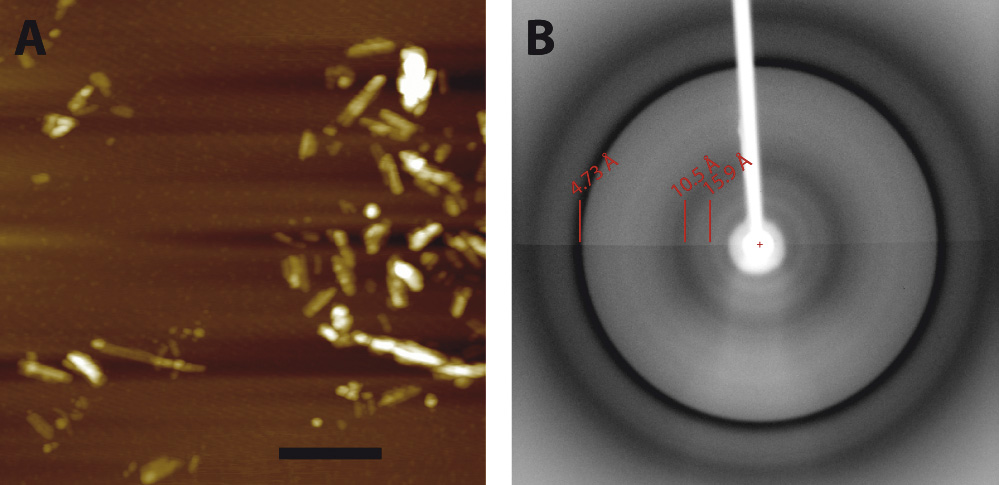
**MoPrP(89-230) fibrils characterisation**. A) Atomic force microscopy images of MoPrP(89-230) amyloid fibrils. The scale bars correspond to 200 nm. B) X-ray fiber diffraction showing prominent sharp reflection at 4.73 Å and diffuse reflection at 10.5 Å.

We have also tested if the soluble MoPrP(89-230) can form fibrils. It is commonly accepted in the prion biology field that PrP amyloid fibrils generated *in vitro *can be used as a synthetic surrogate for PrP^Sc ^[23]. We generated *in vitro *the amyloid aggregates using the soluble MoPrP(89-230) because this truncated MoPrP has been extensively studied in fibrils studies [23]. To generate *in vitro *fibrils we used the amyloid seeding assay (ASA) protocol, which is a high-throughput technique for prion fibrils formation *in vitro *developed in the Prusiner laboratory. The fibrilization reaction was performed in quasi-native condition at physiological pH and in the presence of a low concentration of denaturant (0.4 M GuHCl). The polymerization process was monitored simply by applying thioflavin T in the reaction mixture. This dye shows strong increase of fluorescence upon binding to β-sheet rich structures like amyloid aggregates. When used in conjunction with multi-well plates and automated fluorescence plate readers, the ThT represents a feasible, highly sensitive, high-throughput approach for the detection of conformational changes of proteins. In our experiments we observed a strong increase in fluorescence, which corresponds to the ThT binding to β-sheet rich structures like amyloid aggregates (Figure 9). Our results are consistent with previous fibrilization experiments performed with MoPrP(89-230) expressed in *E. coli *as inclusion bodies [28,29] and provide a good indication about the ability of soluble MoPrP(89-230) to form ThT-positive fibrils. Furthermore, to gain insight into the morphology and structure of amyloid fibrils formed using solubly expressed MoPrP(89-230) we performed atomic force microscopy and X-ray fibril diffraction (Figure 10). The observed fibrils were hundreds of nanometers in length and had a measured height and width of approximately 5 nm. Their diffraction pattern shows reflections at 4.7 Å and ~10 Å, characteristic of the cross-β pattern previously observed for fibrils formed by refolded recombinant PrP90-231 [30] and as well as other amyloidogenic proteins [31].

## Conclusion

The formation of the disulfide bond between Cys179 and Cys214 is essential to obtain a correctly folded PrP [12,32]. The human Quiescin Sulfhydryl OXidase (QSOX) allows the formation of native disulfide bonds in eukaryotic proteins expressed in the cytoplasm of *E. coli *[14,33]. We showed that co-expressing PrP (full-length or truncate MoPrP and HuPrP) with QSOX produces a significant amount of correctly folded PrP in the cytoplasm and that a disulfide bond is present in the purified protein. Previous studies showed that co-expression with several chaperones did not succeed in producing reasonable quantities of soluble PrP [8]. Furthermore, soluble but partially degraded PrP could only be produced in the periplasm [9]. In *E. coli*, disulfide bond formation occurs in the periplasm [34]. Co-expression with QSOX is a good alternative to produce mammalian proteins containing disulfide bonds in the *E. coli*.

To the best of our knowledge, there is no evidence for direct interaction *in vivo *of QSOX with the PrP; therefore we believe that QSOX could be used to express different mammalian PrP in *E. coli*. The described method is a simple and effective method for producing large quantities of soluble PrP in *E. coli*, which can subsequently be used for functional and structural studies.

## Competing interests

The authors declare that they have no competing interest

## Authors' contributions

RA, HH, WG, SR, GG and MA performed the experiments. AW prepared the graphic files. JM, SS, GL, JS and AW conceived and designed the experimental approach. AW coordinated the whole study. SR, SS, JM, AW prepared the manuscript. RA, HH cloned, expressed and purified the recombinant PrPs. SR performed NMR. WG performed the thiostar assay. GG performed ASA assay. MA performed AFM and fiber diffraction. All authors read and approved the final manuscript.
